# Scientific

**Published:** 1889-10

**Authors:** 


### SCIENTIFIC ITEMS.

**The Conflict** between the electricians in regard to the power of the current to produce instant death, when used for the execution of criminals, shows no signs of abating; but, meanwhile, preparations for the execution of the condemned murderers in New York are almost completed. The whole matter has been purposely enveloped in a mist of uncertainty by rival electric light companies, petty politicians, and those misguided sympathizers with criminals, who are almost as dangerous to society as the criminals themselves. There is not the slightest reason, scientific or practical, why a current of electricity cannot easily be produced of a strength sufficient to instantly kill the person through whose body it is sent, and, as the speed of electricity is greater than the passage of a sensation along the nerves, it is probable that such a death would be absolutely painless—although that is not an especially important matter, when we consider the lack of enterprise of the average murderer in devising painless methods of death for his victims.—*Pop. Science News.*

**Experiments** with Brown-Sequard's alleged elixir of life continue to be reported in the daily papers, with varying results, but no reliable tests have yet been published in the medical journals. We consider the whole matter a piece of sensational rubbish, entirely unworthy the attention that has been given it, and predict that before many days the new elixir will have gone to that bourne where Bergeron's sulphuretted hydrogen injections, and many similar discoveries, now rest in peaceful oblivion. As an instance of the lack of scientific knowledge of certain experimenters, we note that one physician carefully *sterilized* the fluid before injection. We presume the same train of reasoning would lead him to heat vaccine lymph above the boiling point before performing the operation of vaccination.—*Ib.*

**An Automatic Reading Lamp.**—The latest and most ingenious application of the penny-in-the-slot automatic apparatus is the adaptation of this principle to the supply of electric light in the shape of reading lamps for railway carriages, omnibuses, tramcars, etc. The lamp in question has been patented by a Leeds firm. The lamp, as originally patented, consists of a clockwork apparatus, contained in a box 5in. by 5in. and 3in. And by introducing a penny into the top of the machine, and subsequently pressing a knob, the mechanism is set in motion, and an electric light obtained, which, after burning for half an hour, is automatically extinguished. The lamp can, moreover, be extinguished at will, by pressing a second knob. This lamp will, it is stated, be in use in the course of a few days on the Great Western Railway. The lamps are lighted from an accumulator, which, placed in any of the compartments of a carriage, will supply with electricity the whole of the lamps in the carriage. The accumulator can thus be charged at any station without interfering with the light. The accumulators will be charged with a forty hours supply. A remarkable feature is that should the lamp fail to act, the coin deposited is automatically returned to the operator. The lamps will be fixed under the hat rail of the carriages, and, by this means, passengers can, when leaning back, read at their ease.—*Mechanical World* (*Eng.*)

**The Mosquito.**—To expel mosquitoes, take of gum camphor a piece about one-third the size of a hen's egg, and evaporate it by placing it in a tin vessel and holding it over a lamp, taking care that it does not ignite. The smoke will soon fill the room and expel the mosquitoes, and they will not return, even though the windows should be left open all night.—*The Doctor.*

I learned the secret of successful warfare against these pests when living in the swamps of Louisiana, where, in summer or winter, mosquitoes swarm. For some years life was unendurable, and no meal could be eaten in peace. But all at once there was a change for the better. Bars and screens were often out of place, but there was almost an immunity from insects. I had just changed my colored boy. The new comer explained how he kept the "critters" away. He burned small pieces of gum camphor on the cook stove, and used a secret preparation he called "sudekillo." When I married and came to Missouri- I imparted the secret to my wife, and as there is no patent on it that I know of, I would advise all fellow sufferers to go and do likewise. The gum camphor alone is ample for the purpose, and need only to be used two or three times a week.—*St. Louis Globe Dem.*

**Sixty years ago** railroads were unknown in this country, and the population of the United States consisted of 12,000,000 people. To day we operate upward of 165,000 miles of railroad, and our population has increased to 60,000,000. Sixty years ago the aggregate wealth of the United States was less than $1,000,000,000; at present it is estimated at $56,000,000,000. Over our 165,000 miles of railroad there was carried last year 47,000,000 people, and 600,000,000 tons of freight were transported. Upon these lines are engaged 1,000,000 employes. Their equipment consists of 30,000 locomotives, 21,000 passenger cars, 7,000 baggage cars, and 1,000,000 freight cars. The capital invested in construction and equipment amounts to $8,000,000,000, and the yearly disbursements for labor and supplies exceed $600,000,000.

**Magnesium** is one-third lighter than aluminum, at the same time more dense, harder, and tougher. An article made from German silver weighing 5.5 kg. weighs only 1 kg. if made of magnesium. Atmospheric influence is about the same on magnesium and aluminum, but while alkalies, such as ammonia or soda, attack aluminum considerably, magnesium is not effected by them at all. Magnesium is worked into objects having sharp edges, screws, etc., more readily and with better results. It takes a high polish, is readily hammered and rolled; can be swaged or pressed like tin into any shape. It is at present about one-fifth cheaper than aluminum.

